# Differences in internalization and growth of *Escherichia coli* O157:H7 within the apoplast of edible plants, spinach and lettuce, compared with the model species *Nicotiana benthamiana*

**DOI:** 10.1111/1751-7915.12596

**Published:** 2017-02-07

**Authors:** Kathryn M. Wright, Louise Crozier, Jacqueline Marshall, Bernhard Merget, Ashleigh Holmes, Nicola J. Holden

**Affiliations:** ^1^ Cell and Molecular SciencesThe James Hutton InstituteDundeeUK

## Abstract

Internalization of food‐borne bacteria into edible parts of fresh produce plants represents a serious health risk. Therefore, internalization of verocytotoxigenic *E. coli* O157:H7 isolate Sakai was assessed in two species associated with outbreaks, spinach (*Spinacia oleracea*) and lettuce (*Lactuca sativa*) and compared to the model species *Nicotiana benthamiana*. Internalization occurred in the leaves and roots of spinach and lettuce throughout a 10 day time‐course. The plant species, tissue type and inoculum dose all impacted the outcome. A combination of low inoculum dose (~10^2^ CFU) together with light microscopy imaging highlighted marked differences in the fate of endophytic *E. coli* O157:H7 Sakai. In the fresh produce species, bacterial growth was restricted but viable cells persisted over 20 days, whereas there was > 400‐fold (~2.5 Log_10_) increase in growth in *N. benthamiana*. Colony formation occurred adjacent to epidermal cells and mesophyll cells or close to vascular bundles of *N. benthamiana* and contained components of a biofilm matrix, including curli expression and elicitation, extracellular DNA and a limited presence of cellulose. Together the data show that internalization is a relevant issue in crop production and that crop species and tissue need to be considered as food safety risk parameters.

## Introduction

Verocytotoxigenic *Escherichia coli* (VTEC) is a food‐borne pathogen that can cause serious disease ranging from haemorrhagic colitis to life‐threatening haemolytic uraemic syndrome (HUS) and central nervous system damage (Kaper *et al*., 2004). Although most cases were previously associated with contamination of meat and milk products, in recent years fresh fruit and vegetables have been increasingly implicated as the sources of infection and on an international scale, ~20% of food‐borne VTEC outbreaks are thought to have arisen from fresh produce (Greig and Ravel, 2009). One of largest outbreaks of VTEC occurred in Japan in 1996, with almost 10 000 cases and 12 deaths, as a result of contamination of white radish sprouts with *E. coli* O157:H7 (Michino *et al*., 1999; Watanabe *et al*., 1999). The isolate responsible, *E. coli* O157:H7 Sakai, forms the basis of the current study. Whilst transient surface contamination of fresh produce has been demonstrated, it is now recognized that VTEC are able to colonize plants as secondary hosts in a manner that has some key differences from their primary host, cattle (Holden *et al*., 2009). Indeed, specific *E. coli* isolates appear to have adapted to their hosts with isolates in phylogenetic group B1 more commonly associated with plants, whereas phylogroups A and B2 are linked to an animal‐associated lifestyle (Méric *et al*., 2012).

VTEC and other food‐borne pathogens, such as *Salmonella enterica*, can exist on both external and internal tissues of plants (Deering *et al*., 2012; Erickson, 2012; Hirneisen *et al*., 2012; Hou *et al*., 2013; Wright *et al*., 2013; Martinez *et al*., 2015). This capability presents a food safety threat in crop production, as internalized bacteria cannot be removed with standard sanitation practices, although treatments such as irradiation, ultrasound and cold plasma can be effective (Gomes *et al*., 2009; Bilek and Turantaş, 2013; Ziuzina *et al*., 2015). Internalization into plants is defined as the ability of bacteria to penetrate into the internal tissues, where the bacteria normally reside in the extracellular spaces of the apoplast (Godfrey *et al*., 2010). Some well‐characterized phytopathogens also reside close to the leaf surface in the stomatal pores (Yu *et al*., 2013), and other plant‐associated bacteria are known to internalize (Turner *et al*., 2013). Internalization of *E. coli* has been demonstrated by recovery of bacteria following surface sterilization of the plant tissue (Solomon *et al*., 2002; Warriner *et al*., 2003b; Gomes *et al*., 2009; Deering *et al*., 2011; Hirneisen *et al*., 2012; Wright *et al*., 2013; Erickson *et al*., 2014).

We have previously demonstrated that a large proportion (81–91%) of spinach plants maintained in hydroponic culture support an internal population of bacteria, which accounted for ~0.5% of the total population (Wright *et al*., 2013). Within the roots, these bacteria were located inside the cell walls of epidermal and cortical cells and within the apoplast between the plant cells. Similarly, colonization and invasion of five‐ to 7‐day‐old *Arabidopsis thaliana* roots by *E. coli* O157:H7 strain Odwalla were observed along with proliferation of bacteria on the roots and shoots over a 3 day period (Cooley *et al*., 2003). Young lettuce seedlings grown in soil containing *E. coli* O157:H7‐spiked manure were colonized on the surface, with some bacteria also located within the leaves (Solomon *et al*., 2002). As a second route of contamination, spiked irrigation water applied to the roots yielded bacteria from the mature leaves (Solomon *et al*., 2002). However, the route by which bacteria migrate from roots to shoots remains unclear, particularly in relation to whether bacteria can move through the vasculature.

Occurrence of internalization under conditions that are relevant to horticulture and at ecologically appropriate inoculum levels has been demonstrated, although it appears to occur at a low frequency (Erickson *et al*., 2010, 2013). This raises questions relating to the internalization ability of food‐borne pathogens and its relevance in food safety, i.e. do sufficiently high enough numbers of bacteria get to and stay within the plant material to pose a food safety threat to the consumer at the end of the food chain? More specifically, how well do VTEC internalize; how do internalized VTEC fare over prolonged time periods; does any specificity exists in the plant–microbe interactions or is the interaction generic among hosts? As such, the aim here was to determine the ability and subsequent fate of the most common VTEC serotype, O157:H7 (isolate Sakai), to internalize into edible species that have been associated with food‐borne VTEC outbreaks, as well as a well‐characterized member of the *Solanaceae*,* Nicotiana benthamiana*, grown under commercially relevant conditions and to harvestable age. Use of *N. benthamiana* allowed utilization of genetic resources that are not available for edible species. Finally, the growth rates of internalized *E. coli* O157:H7 were measured using a very low density of starting inocula (tens of CFU) and the molecular nature of apoplastic colonies described.

## Results

### Variation in the ability of *E. coli* Sakai to internalize into different hosts and tissues

In our previous work, we found that *E. coli* O157:H7 Sakai internalized into the roots and leaves of spinach and lettuce grown under hydroponics conditions (Wright *et al*., 2013), but the rate at which this occurred for plants grown under conditions more relevant to commercial production is unknown. Thus, the ability of *E. coli* Sakai to internalize was measured in the roots and leaves of spinach and lettuce grown in compost, in a glasshouse. Two different inoculum levels were used: a high dose at 10^7^ CFU ml^−1^ that is appropriate for laboratory‐scale assessments of bacteria–plant interactions and microscopy, and at a lower dose, 10^3^ CFU ml^−1^ that is more likely to be found in nature from contaminated soil or irrigation water (Matthews *et al*., 2006). Internalization was defined as the number of bacteria recovered from plant tissue following surface sterilization with 200 ppm calcium hypochlorite for 15 min. These levels, which are ~2.5 times higher than that used in industrial production for maintaining a degree of cleanliness of produce washing water (Ramos *et al*., 2013), were used to keep the work relevant to vegetable production. A large level of variation in the efficiency of surface sterilization occurred, ranging from 38% for spinach leaves inoculated with a high dose, to 100% for lettuce roots or leaves inoculated with a low dose of bacteria (Table 1). Incomplete surface sterilization meant that it was not always possible to obtain a full data set, e.g. for lettuce leaves inoculated with a high dose of bacteria, and indicated that higher concentrations of hypochlorite are required for complete removal of surface‐associated *E. coli* Sakai.

**Table 1:** Counts of *E. coli* Sakai recovered from spinach, lettuce and *N. benthamiana* tissue

Tissue^a^	Dose^b^	Time (days)	Positive plants^c^ (*n*)	Total counts^d^ (log_10_)	Internal counts^d^ (log_10_)	Internal population^e^ (%)	Sterilis. efficiency^f^ (%)
*Spinach var. Amazon*							
Root	10^7^	0	9 (10)	5.42 ± 0.20	2.31 ± 0.75	0.08	
		5	10 (11)	5.12 ± 0.37	2.62 ± 0.92	0.31	
		10	12 (14)	4.29 ± 0.67	2.37 ± 1.15	1.21	80
	10^3^	0	5 (14)	1.45 ± 0.67	0.53 ± 0.79	31.43	
		5	1 (15)	0.82 ± 0.79	0.08 ± 0.29	3.60	
		10	4 (15)	1.17 ± 1.34	0.21 ± 0.44	0.17	97.7
Leaf	10^7^	0	1 (1)	5.14 ± 0.38	3.66 ± N/A	2.28	
		5	4 (6)	3.51 ± 1.41	0.99 ± 0.85	0.08	
		10	9 (10)	3.81 ± 1.09	1.81 ± 1.06	1.28	37.7
	10^3^	0	12 (12)	2.53 ± 2.71	1.73 ± 1.81	16.09	
		5	2 (10)	1.15 ± 1.45	0.27 ± 0.59	13.27	
		10	5 (13)	1.98 ± 2.44	1.50 ± 1.85	32.56	77.7
*Lettuce var**.** AYR*							
Root	10^7^	0	10 (10)	4.85 ± 0.43	2.19 ± 0.55	0.22	
		5	7 (10)	4.36 ± 0.54	2.17 ± 0.81	0.65	
		10	10 (13)	3.98 ± 0.35	1.87 ± 0.70	0.78	78.3
	10^3^	0	11 (15)	1.73 ± 0.63	1.33 ± 0.89	39.53	
		5	11 (15)	1.69 ± 1.09	1.02 ± 0.67	21.42	
		10	5 (15)	2.11 ± 0.78	0.94 ± 1.40	6.82	100
Leaf	10^3^	0	11 (15)	1.84 ± 0.40	0.81 ± 0.53	9.30	
		5	14 (15)	1.87 ± 1.02	1.77 ± 0.87	78.27	
		10	9 (15)	2.04 ± 1.95	0.92 ± 0.88	7.61	100
*N. benthamiana*							
Leaf	10^3^	0	10 (15)	1.89 ± 0.65	0.86 ± 0.69	9.23	
		5	9 (14)	2.24 ± 1.20	1.66 ± 1.34	26.28	
		10	12 (15)	1.78 ± 1.58	1.34 ± 0.98	36.15	97.7

**a.** Plant roots were inoculated via the pot‐soak method for 1 h, or the leaves dipped into a bacterial suspension for 30 s.

**b.** The inoculum dose of *E. coli* Sakai expressed as CFU ml^−1^.

**c.** The number of plants containing internalized bacteria, along with the total number of plants assessed (experiments were repeated three times with a maximum of five replicate plants, giving a possible maximum of 15, although due to poor sterilization efficiency the usable number was sometimes lower).

**d.** The average counts for the total (i.e. untreated) or just internalized population (i.e. surface‐sterilized plant tissue) is given, ± the SD.

**e.** The population of *E. coli* Sakai recovered from surface‐sterilized leaves, expressed as a proportion of the total population.

**f.** The efficiency of surface sterilization, expressed as a percentage of samples that did not contain detectable *E. coli* Sakai on the external surface, post‐surface sterilization. The data are from an average of all time points and biological replicates.

Recovery of internalized *E. coli* Sakai varied widely between the plant species, tissue type and starting inoculum, although internalized bacteria were recovered in every case and for each time point (Table 1). Inoculation with the high density (10^7^ CFU ml^−1^) resulted in ~5 log_10_ CFU total population on roots or leaves for the starting time point, whereas inoculation at the low density (10^3^ CFU ml^−1^) resulted in ~2 log_10_ CFU total population for the same point. Inoculation at the higher density resulted in the highest number of internalized *E. coli* Sakai, with the maximum numbers found in spinach roots 10 dpi (2.37 log_10_ CFU), followed by lettuce roots (1.87 log_10_ CFU) and spinach leaves (1.81 log_10_ CFU), at the same time point. However, there were no significant differences between any of the groups at this dosage, based on presence/absence analysis. The lowest numbers recovered occurred for the lower inoculum at 5 dpi in spinach roots (0.08 log_10_ CFU) and spinach leaves (0.27 log_10_ CFU), although in both cases, the numbers increased by 10 dpi (to 0.21 and 1.50 log_10_ CFU respectively). The numbers of internal *E. coli* Sakai were significantly higher in lettuce roots compared with spinach roots, determined by an unbalanced anova on presence/absence counts (*P* < 0.05) for the low dosage, and there was a significant interaction between the mean responses for plant species and tissue type (*P* < 0.01). The highest proportion of internalization occurred at 10 dpi in the leaves of *Nicotiana benthamiana* inoculated with a low dose, with 36% of the total population (i.e. external + internal) internalized (1.34 log_10_ CFU). At the low dose, regression analysis showed that there were significant differences for the total populations of bacteria present, between both plant species (*P* < 0.01) and for a plant species by tissue interaction (*P* < 0.05). The increase in the total population on the leaves of lettuce and the roots of both lettuce and spinach inoculated at a low dose was indicative of proliferation of the bacteria on external tissue.

### Stability of the reporter plasmid in internalized *E. coli* O157:H7 Sakai

The extent of variation in internalization ability showed, to some extent, a plant species and tissue type influence. Therefore, we used microscopy to examine the fate in internalized *E. coli* Sakai in more detail. The bacteria were transformed with a plasmid‐borne GFP reporter, under the control of the *E. coli gyrA* promoter (termed *E. coli* Sakai‐GFP), to aid in detection. The long‐term maintenance of the plasmid‐borne GFP reporter was found to be stable for at least 10 days following examination of *E. coli* Sakai‐GFP drop inoculated onto the leaves of spinach or *N. benthamiana* leaves. There were no significant differences in the total population (i.e. external and internal) of *E. coli* Sakai‐GFP recovered on agar either lacking or containing the selective antibiotic from *N. benthamiana* (average of 6.09 or 6.13 log_10_ CFU, respectively) or spinach leaves (average of 4.47 or 4.56 log_10_ CFU respectively).

### Colony development by internalized *E. coli* Sakai‐GFP in *N. benthamiana* leaves

**Figure 1:**
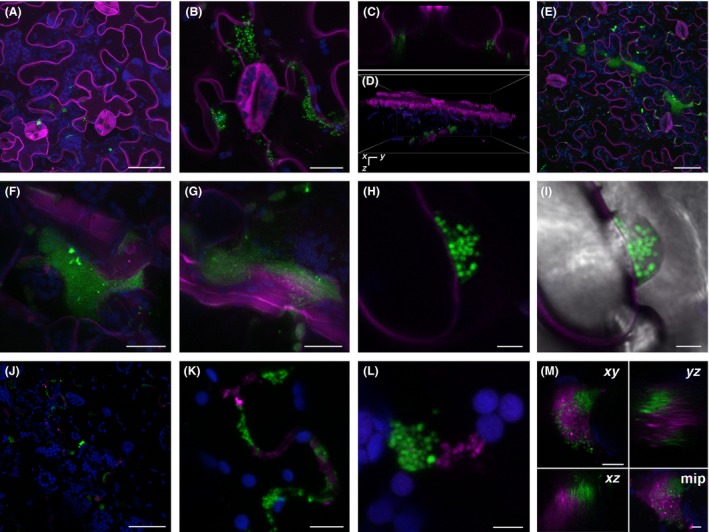
Colonization of *Nicotiana benthamiana* leaves by *Escherichia coli* O157:H7 Sakai. (A) Maximum intensity projection of abaxial epidermal and mesophyll cells of mOrg‐LTI‐benth (magenta) observed 4 dpi with 10^7^ CFU ml^−1^ Sakai‐GFP (green) showing colonies formed within the apoplast and chloroplasts (blue) mainly in the mesophyll cells. (B) Maximum intensity projection 23 dpi of larger colonies located at the junction between epidermal cells and (C) an orthogonal yz projection. (D) 3D and (E) maximum intensity projection of a z‐stack 143 μm deep showing colonies adjacent to bundle sheath cells, 23 dpi. Two of these colonies at higher magnification: (F) a maximum intensity projection of a colony at least 35 μm deep and (G) 39 μm deep located around 70–80 μm below the epidermal surface. Single confocal section taken of a colony 23 dpi showing (H) individual *E. coli* Sakai‐GFP bacteria (green) in a colony adjacent to the plasma membrane of an epidermal cell (magenta) with a surrounding structure (I) visible using the transmitted light detector. Maximum intensity projections of the abaxial face of a *N. benthamiana* leaf 19 dpi with a combination of *E. coli* Sakai‐GFP (green) and *E. coli* Sakai‐RFP (magenta), with colony formation in the apoplastic spaces (J) and at junctions between adjacent epidermal cells (K, L). Separate‐coloured colonies were associated with a surrounding matrix (M) here seen as single xy, xz, yz sections or as a maximum intensity projection (mip). Scale bars 50 μm (A, J), 20 μm (B–G), 10 μm (K, L), 5 μm (H, I, M).

Once it was established that the GFP reporter could be used to detect *E. coli* Sakai, it was possible to characterize the fate of internalized bacteria. *E. coli* Sakai was infiltrated into plant leaves to investigate its fate in isolation of epiphytic bacteria. Bacteria for plant inoculations were cultured at 18°C to avoid any temperature‐shifts, as temperature is a key environmental factor known to affect *E. coli* gene expression (Crozier *et al*., 2016) that could in turn, affect the fate of internalized bacteria. *E. coli* Sakai‐GFP were infiltrated into *N. benthamiana* leaves at the high dose (10^7^ CFU ml^−1^) to ensure that there were sufficient fluorescent cells visible for examination by microscopy. *N. benthamiana* expressing a fluorescent plasma membrane reporter (termed mOrg‐LTI‐benth) was used to aid with *in planta* localization of *E. coli* Sakai‐GFP. Four days of postinfiltration, the bacteria were observed in colonies frequently associated with the internal face of the abaxial epidermal cells in the channels between adjoining cells (Fig. 1A). At a later time point, 23 dpi, increased sized colonies were located associated with the epidermis (Fig. 1B–C). Colonies were also visible at a much greater depth into the tissue, associated with the bundle sheath cells (Fig. 1D–G). All *E. coli* Sakai‐GFP were observed to be in the apoplastic space of the leaf, with no indication of penetration by the bacteria into the plant cells. Some individual bacterial cells within the colonies could be distinguished (Fig. 1H) along with a fluorescent matrix, which in some cases could also be detected using transmitted light (Fig. 1I).

To determine whether development of colonies of infiltrated *E. coli* Sakai‐GFP had arisen as a result of bacterial proliferation, wild‐type (i.e. non‐labelled) *N. benthamiana* were infiltrated with a mixture of *E. coli* Sakai transformed with either the GFP reporter or a RFP reporter. After 19 days, separate GFP‐ and RFP‐expressing colonies were detected at the internal boundary of the epidermal cells (Fig. 1J–L), with larger sized colonies associated with the mesophyll cells (Fig. 1M). The lack of mixing of differently labelled bacterial cells indicates that colonies developed from proliferation of a single GFP‐ or RFP‐expressing bacterium.

### Internalized *E. coli* Sakai‐GFP do not form colonies in spinach, lettuce or tomato leaves

**Figure 2:**
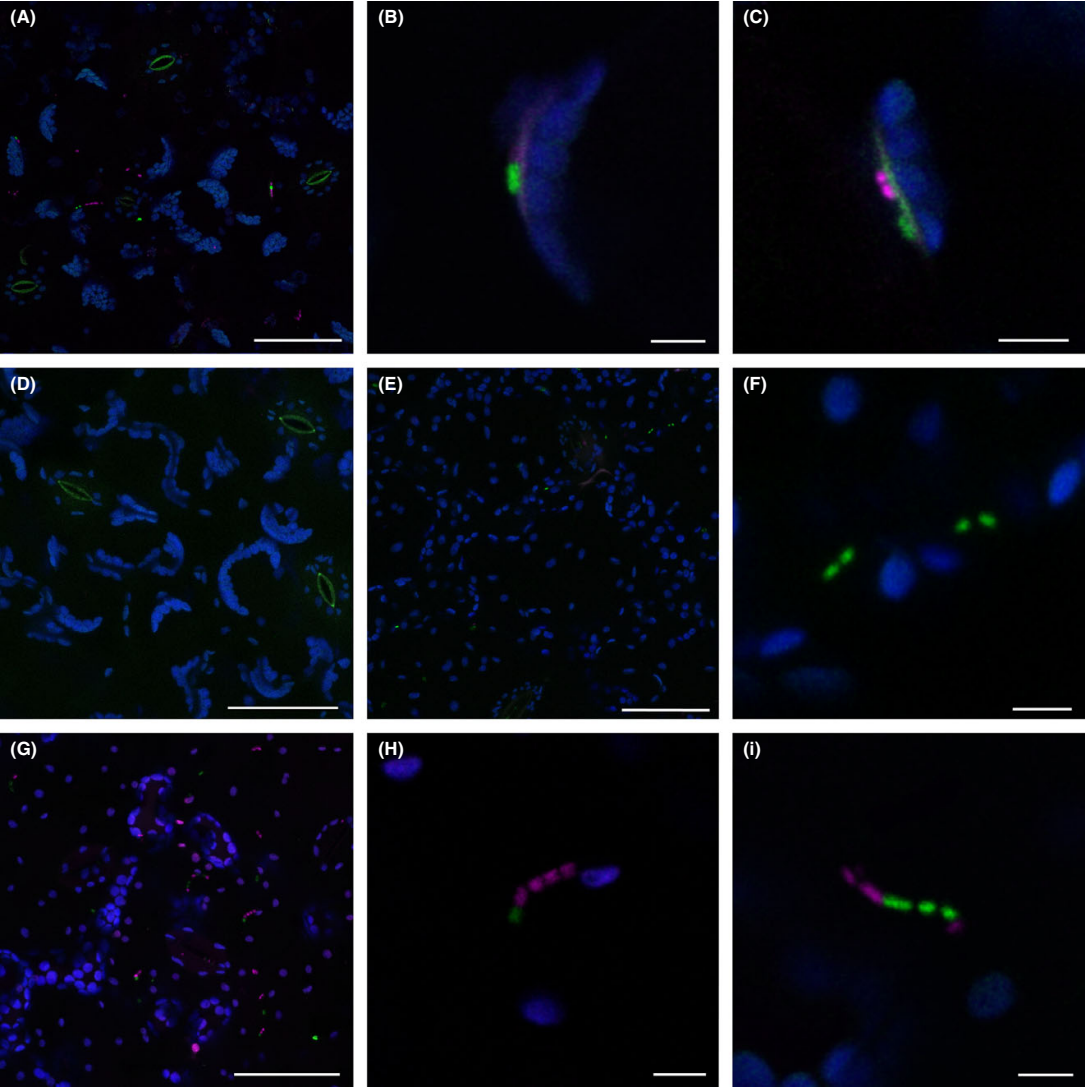
Colonization of spinach, lettuce or tomato leaves by *Escherichia coli* O157:H7 Sakai. *E. coli* Sakai‐GFP (green) and *E. coli* Sakai‐RFP (magenta) co‐infiltrated into the apoplast of a spinach leaf 14 dpi (A–C) and tomato 21 dpi, (G–I), or *E. coli* Sakai‐GFP infiltrated into lettuce 4 dpi (E–F). An uninoculated control of a spinach leaf infiltrated with 0.5 × MS buffer 13 dpi (D). Maximum intensity projections (A, D, E, G) or single sections (B, C, F, H, I) are shown. Scale bars 50 μm (A, D, E, G), 5 μm (B, C, F, H, I).

To compare the fate of internalized *E. coli* Sakai in edible crop species associated with outbreaks of food‐borne pathogens, infiltrated bacteria were examined in the leaves of spinach, lettuce or tomato. Infiltration of a combination of *E. coli* Sakai‐GFP or Sakai‐RFP resulted in a different pattern of colonization to that seen in *N. benthamiana*. Even after prolonged incubation of 14–21 days, only a few bacteria were detected within spinach (Fig. 2A). On frequent occasions, a single bacterium was observed attached to mesophyll cells adjacent to a region of autofluorescence within the cell wall (Fig. 2B). Such regions were not observed in tissue infiltrated with MS buffer‐only negative control (Fig. 2D). Small chains of *E. coli* Sakai cells, grouped by the GFP or RFP reporter (Figs 2C, F and H–I), were indicative of limited proliferation in all three species.

**Figure 3:**
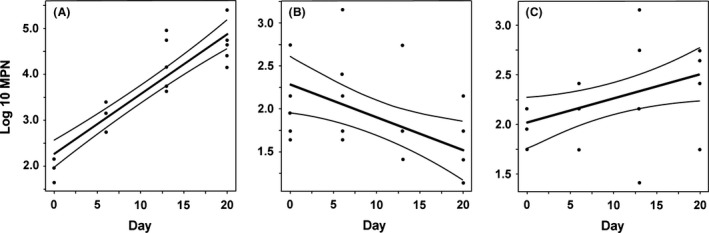
Growth or persistence of endophytic *E. coli* Sakai. Endophytic *E. coli* Sakai‐GFP recovered from leaves of (A) *Nicotiana benthamiana*, (B) spinach or (C) tomato was estimated by MPN. Individual data points are plotted for replicate plants and the slope of the curve fitted (straight lines, middle), bounded by 95% confidence limits (upper and lower lines). For each plant species, six replicate samples were assessed at each time point. The data represent one experimental replicate (see Table 2 for others).

The lack of *E. coli* Sakai colony development in the apoplast of spinach, lettuce or tomato compared with *N. benthamiana* indicated large differences in the ability of internalized bacteria to grow. Therefore, we determined the *in planta* growth rate for *E. coli* Sakai‐GFP. Very low inoculum densities of ~20 to 50 cells were used to minimize background ‘noise’ from cell turnover and thus allow growth to be definitively measured. This necessitated the use of MPN to estimate numbers below the limit of detection by direct plating. The numbers of *E. coli* Sakai‐GFP infiltrated into the leaves of *N. benthamiana* significantly increased during the first four sampling times, up to 15–21 dpi (Fig. 3) although there was variation between individual plant replicates (Table 2). Growth between d1 and d14 was in the order of 10.5 generations, which represents a generation time of ~1.3 days. A similar growth rate was measured with a higher starting inoculum, suggesting that growth was not limited by nutrient availability within the apoplast (Table 2). In contrast, infiltration of *E. coli* Sakai‐GFP into spinach or tomato leaves showed either minimal or no increase in the bacterial population (Fig. 3B–C). Infiltration into lettuce leaves generated large variation in the numbers of bacteria recovered (data not shown), possibly due to technical variation in the success of infiltration compared with the other species, and thus in our hands it was not possible to obtain a sufficiently robust growth rate for this species. Therefore, tomato leaves were included as a second fresh produce crop: although tomato leaves are not eaten, VTEC has been recovered from retail tomato fruits (Gomez‐Aldapa *et al*., 2013) and the related food‐borne pathogen *S. enterica* is often linked to their consumption (Bennett *et al*., 2015).

**Table 2:** Linear regression analysis of internalized *E. coli* Sakai recovered from the three plant species, as determined by MPN log_10_

Trial	Expt. No.^a^	Inoculum Dose	Growth (log_10_ MPN/day)	Standard error	Significance
*N. benthamiana*	1	10^3^	0.244	0.022	< 0.001
	2	10^3^	0.132	0.023	< 0.001
	3	10^3^	0.130	0.012	< 0.001
	3	10^5^	0.145	0.015	< 0.001
Spinach	2	10^3^	0.004	0.018	NS
	3	10^3^	−0.038	0.013	0.007
Tomato	4	10^3^	0.039	0.020	NS
	5	10^3^	0.024	0.010	0.025

**a.** The numbers refer to different, independent experiments that were repeated to assess growth or persistence *in planta*. Some experiments with different species were run concurrently in the plant growth cabinet, e.g. #2 or #3; the rest were done at separate times.

### Characterization of *E. coli* Sakai apoplastic colonies in *N. benthamiana* leaves

**Figure 4:**
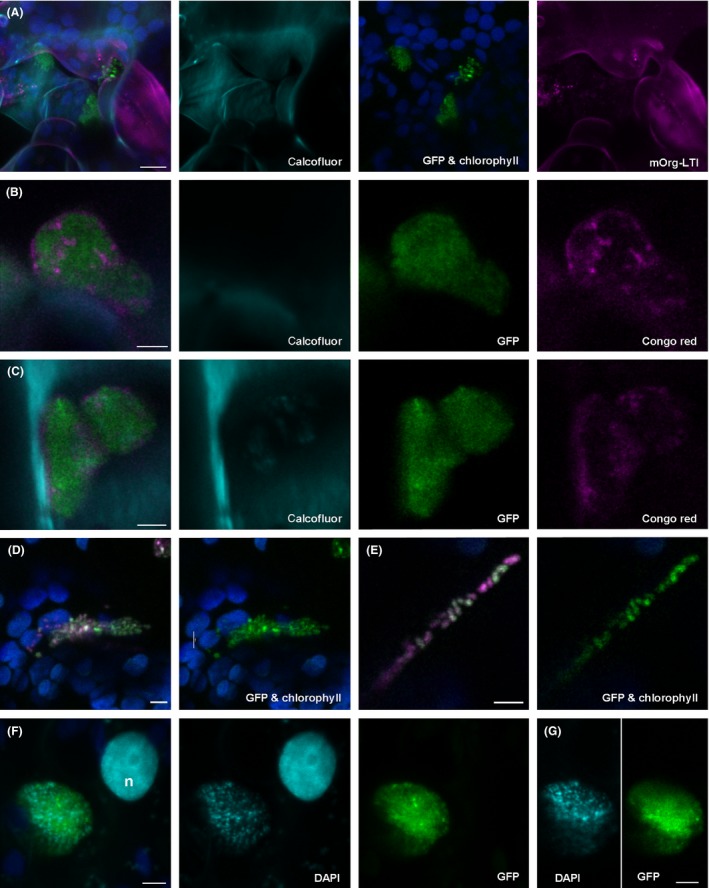
Characterization of *E. coli* Sakai biofilm within the *Nicotiana benthamiana* leaf apoplast. Confocal fluorescence microscopy of *N. benthamiana* infiltrated with *E. coli* Sakai, showing different colour channels (indicated) or a merged image (left‐hand side). (A) Maximum intensity projection of colonies (green) 13 dpi in a mOrg‐LTI‐benth leaf. Cellulose in the plant cell walls is stained with calcofluor (cyan) and is external to the plasma membrane (magenta) surrounding chloroplasts (blue) within the cell. Single sections taken of *E. coli* Sakai‐GFP colonies 20 dpi in a *N. benthamiana* leaf with (B and C) staining of the periphery and some internal structures with Congo red. Maximum intensity projection (D) and single section (E) of *E. coli* Sakai cotransformed with *csgBA‐gfp+* and *gyrA‐rfp* 5 dpi in a *N. benthamiana* leaf. Chloroplasts are false‐coloured blue. Maximum intensity projection (F) and single section (G) of an *E. coli* Sakai‐GFP colony (green) stained with DAPI (cyan) adjacent to a nucleus (n), 15 dpi in a *N. benthamiana* leaf. Scale bars 10 μm (A), 5 μm (B–G).

Development of *E. coli* Sakai‐GFP colonies within the apoplast of *N. benthamiana* were often accompanied by an unknown matrix (Figs 1I and 4A). As colonization of plants by enteric pathogens has been reported to be, in part, dependent on the ability to produce the biofilm components curli (Macarisin *et al*., 2012; Hung *et al*., 2013) and cellulose (Barak *et al*., 2007; Carter *et al*., 2011), it was possible that the VTEC matrix *in planta* also contained these components. Therefore, indicator dyes, calcofluor white for cellulose and Congo red for curli, together with a plasmid‐borne curli reporter fusion (*csgBA‐gfp*), were used for their detection on well‐developed *E. coli* Sakai‐GFP apoplastic colonies in *N. benthamiana* or mOrg‐LTI‐benth. Treatment with either calcofluor white or Congo red did not result in staining of *E. coli* Sakai prior to infiltration indicating that neither component was expressed to detectable levels during *in vitro* growth (Fig. S1). However, Congo red staining occurred on the periphery and some internal structures of the apoplastic *E. coli* Sakai‐GFP colonies Fig. 4B and C). Expression of the curli structural gene promoter, *csgBA*, was also observed in apoplastic *E. coli* Sakai, (Fig. 4D and E). Prior to infiltration, *E. coli* Sakai was grown in *in vitro* conditions (RD‐MOPS glucose at 18°C) that repressed *csgBA* expression, with only background levels of fluorescence, 67.5 ± 63 RFU, detected from a mid‐log phase culture (OD_600_ of 0.6). In comparison, *in vitro* induction occurred when *E. coli* Sakai was cultured in RD‐MOPS glycerol (287 ± 36 RFU, 95% CI), and to a marginal, but not significant extent in spinach leaf lysates (167 ± 39, 95% CI). Elicitation of curli fibres from *E. coli* O157:H7 Sakai, from Congo red staining, was observed *in vitro* but only when cultured on curli induction medium at 37°C (red colonies) and not at 18°C, or on indicator plates made with M9 glucose (white colonies). Together, the data indicate a specific signal derived from live plants was responsible for curli production in the apoplast biofilms.

Calcofluor white binds cellulose from plants and bacteria non‐discriminatorily, and as expected the majority of staining occurred in plant cell walls, external to the mOrange‐labelled plasma membrane (Fig. 4A). There was only limited evidence of bacteria‐derived cellulose in the biofilm matrix, where the majority of bacterial colonies observed did not stain with calcofluor white (Fig. 4A and B), although staining of internal colony structures that did not coincide with Congo red was observed occasionally (Fig. 4C). Extracellular DNA (eDNA) has also been associated with *E. coli* biofilms (Tetz *et al*., 2009) and staining with DAPI indicated discrete structures within a colony (Fig. 4F and G). Whilst DAPI stained *E. coli* Sakai cells *in vitro* prior to infiltration (Fig. S1C), the apoplastic stained structures did not always coincide with intact bacterial cells within the colony (Fig. 4G). Together, these data demonstrate that curli, eDNA, and to a lesser extent cellulose are components of the biofilm matrix that are expressed in apoplastic *E. coli* Sakai colonies and not under relevant *in vitro* growth conditions.

## Discussion

VTEC are able to colonize plants as secondary hosts, and although the vast majority of the colonizing population exists as epiphytes, a subpopulation can penetrate the internal tissues where they are located in the apoplast. As endophytic behaviour of human pathogens represents a public health threat, there is a need to better understand the ability of the bacteria to internalize and determine their fate over time. The presence of *E. coli* bacteria in the substomatal cavity and in association with the spongy mesophyll has only been demonstrated convincingly in seedlings germinated from inoculated seed (Itoh *et al*., 1998; Warriner *et al*., 2003a; Deering *et al*., 2011). Here, we show some of the dynamics of *E. coli* O157:H7 movement to internal tissue of mature plants of spinach, lettuce and *N. benthamiana* by examining bacterial location over time. One of the key findings was that internalization occurred in every case and at each time point tested, underscoring the propensity of *E. coli* Sakai to invade plant tissue. Both the plant species and tissue type significantly impacted internalization for the low inoculum dose, with the highest level observed in lettuce roots and the lowest in spinach leaves. Others have reported on the presence of endophytic VTEC under a variety of conditions, reviewed in Deering *et al*. (2012) and Hirneisen *et al*. (2012); however, it has not been possible to determine the fate of the bacteria over time. Using a combination of a very low‐cell‐density inoculation and taking advantage of advances in detector sensitivity for deep imaging to vascular tissue, species‐specific differences that affect internalized *E. coli* Sakai have come to light.

The fate of internalized *E. coli* O157:H7 Sakai in spinach or lettuce was distinct from those cells on the external tissue. Higher levels of total colonization (both internal & external) tended to occur on the roots for all species tested. Although roots of these species are not consumed, mechanized harvesters can introduce root tissue, which subsequently requires removal during trimming and packaging, thus running the risk of microbial cross‐contamination to edible tissue (Reed, 2011). In contrast to proliferation of the external population, the numbers of internalized bacteria did not increase in the edible crop species tested [comparison of Table 1 and data in Crozier *et al*. (2016)]. The data were supported by microscopy observations showing apparently arrested/restricted bacterial growth within the leaves of spinach, lettuce or tomato. The occurrence of chains of two to four bacteria indicated limited proliferation, which equated to ~threefold increase in the population in tomato over a 20 day period. A similar pattern of bacterial persistence without multiplication in spinach was reported for *E. coli* O157:H7 14 dpi, although in this case a higher inoculum density was used (Mitra *et al*., 2009). The lack of growth in spinach leaves contrasted with that seen in spinach roots as we previously found endophytic colonization of roots by *E. coli* Sakai‐GFP bacteria, with large numbers of bacteria accumulating within epidermal cells, presumably as a result of proliferation (Wright *et al*., 2013).

In contrast to the situation in edible crop species, infiltration of *E. coli* Sakai into the apoplastic spaces of *N. benthamiana* leaves resulted in establishment of large colonies with an increase of > 400‐fold in the numbers of bacteria after 20 days. Formation of essentially distinct colonies that were labelled with either GFP or RFP precluded the possibility that the colonies arose as a result of bacterial aggregation. The molecular basis to the differences in the ability of *E. coli* Sakai to grow, either in different tissues or in different species, is unknown, although it is likely to be a factor of the plant defence system (Melotto *et al*., 2014).

The colonies formed within *N. benthamiana* exhibited some of the characteristics of biofilm extracellular matrix, curli and in some instances cellulose, which have previously been associated with *E. coli* and *S. enterica* colonization of plants (Eberl *et al*., 2007; Lapidot and Yaron, 2009; Macarisin *et al*., 2012; Yaron and Romling, 2014; Carter *et al*., 2016), as well as eDNA. Curli fibres are adhesive structures that play a role in biofilm formation (Barnhart and Chapman, 2006) via adhesion to both biotic and abiotic surfaces and autoaggregation (Goulter *et al*., 2010). Cellulose production is widespread in bacteria (Römling and Galperin, 2015) and frequently co‐expressed with curli (Hufnagel *et al*., 2015). Curli production was evident from both Congo red staining and reporter expression of the *csgBA* promoter, whilst calcofluor white, which stains β‐polysaccharides with high affinity for cellulose (Adav *et al*., 2010), indicated bacterial‐derived cellulose over and above the strong plant‐derived signals. Production of curli was more frequent than cellulose for *E. coli* O157:H7 Sakai *in planta*, indicating differences in expression profiles. In *E. coli* and *Salmonella enterica*, the expression of curli (*csgBA*) and cellulose (*bcsA*) is co‐activated by the transcriptional activator CsgD (Römling *et al*., 1998; Weber *et al*., 2006). However, intra‐ and interstrain variability shows that cellulose is not an essential biofilm component (Uhlich *et al*., 2006) and for some *E. coli* isolates, cellulose regulation occurs through an alternative mechanism, independent of curli (Da Re and Ghigo, 2006). Neither curli nor cellulose formation was detected in the *E. coli* O157:H7 Sakai cultures immediately prior to infiltration and gene expression of *csgBA* only occurred during colony formation and establishment *in planta*, indicating specific signals for induction that could be plant‐ and/or nutrient‐associated. This supports published findings for *E. coli* (Sakai) (Lim *et al*., 2010) and a comparison of *E. coli* isolates from plants or humans and other mammals, where the plant‐associated strains were significantly more likely to display the rdar morphotype, indicative of a biofilm comprising curli and cellulose (Méric *et al*., 2012). A distinction was also found within the *E. coli* O157:H7 serogroup, where plant‐derived isolates were more likely to express curli under nutrient limitation than animal‐derived isolates (Carter *et al*., 2011). A potential trigger for apoplastic curli production in live plants supports data that show curli‐independent biofilm formation on abiotic surfaces for *E. coli* O157:H7 cultured in spinach leaf lysates (Carter *et al*., 2016). The presence of eDNA has been reported previously in *E. coli* biofilms (Tetz *et al*., 2009) and is an essential component for others such as *Pseudomonas aeruginosa* (Whitchurch *et al*., 2002). eDNA in the *E. coli* O157:H7 Sakai biofilm matrix *in planta* may have occurred via an active secretion mechanism or as a result of cell lysis (Whitchurch *et al*., 2002; Wu and Xi, 2009).

Confocal laser scanning microscopy combined with fluorescent fluorochromes has developed as a valuable method to examine biofilms formed on slides (Lawrence *et al*., 2007; Adav *et al*., 2010; Neu *et al*., 2010), or on leaf surfaces (Rossez *et al*., 2014) and for bacteria derived from leaf surfaces (Carter *et al*., 2016). It has also been used to illustrate colony development of phytopathogens *in planta*, e.g. Misas‐Villamil *et al*. (2013), and alternative approaches have shown a role for biofilm development *in planta* for related members of the *Enterobacteriaceae* (Koczan *et al*., 2011). Here, we were able to exploit technical advances for deep tissue imaging to characterize biofilm components of endophytic *E. coli* Sakai *in planta*. Biofilm formation on the leaf epidermal surface is suggested to protect the bacteria from extreme variations in hydration (Morris and Monier, 2003; Eberl *et al*., 2007) but whether they perform a similar function within the moist environment of the leaf interior, offer protection from components of the plant defence or have identical composition is as yet unknown.

It has been suggested that the a key difference between plant (phyto‐) and human enteric pathogens is that the latter do not show significant multiplication on leaf surfaces of mature plants (Yaron and Romling, 2014). However, the fact that growth can occur under specific conditions such as the germination of sprouts (Cooley *et al*., 2003), on cut surfaces (Wachtel *et al*., 2002), or as demonstrated here in a tissue‐ and species‐specific manner indicates a more complex and dynamic interaction. Plant age is another factor that affects the colonization ability of enteric pathogens (Brandl and Amundson, 2008). Here, relatively mature plants of at least 4 weeks old at the point of inoculation were used, to maintain relevance to the harvestable age for leafy vegetables. To determine the risk of internalization to public health, additional considerations including the dose response need to be considered, which for VTEC is variable but estimated to be below a dose of ~1000 bacteria (Strachan *et al*., 2005). As the bacteria do not proliferate within spinach or lettuce leaves, internalization of at least the minimum infectious dose would be required to cause a threat to public health, notwithstanding the contribution of the epiphytic population. However, in species where growth is not restricted, such as *N. benthamiana*, the occurrence of only a few individual bacteria to penetrate into and proliferate within the leaf apoplast would provide sufficient inoculum to exceed the minimum infectious dose. Whilst the lack of bacterial proliferation in the apoplast of spinach or lettuce leaves is reassuring for public health, it is important to identify the factors involved in the multiplication of bacteria within the *N. benthamiana* apoplast and identify other plant species, particularly those used as uncooked food sources, where similar growth can occur.

## Experimental procedures

### Bacterial strains and growth conditions

*Escherichia coli* O157:H7 Stx‐negative strain Sakai (kanamycin resistant) (Dahan *et al*., 2004) was used for the experimental work and *E. coli* strain AAEC189A (Teunis *et al*., 2004) was used for cloning. Bacteria were routinely cultured ~18 h in lysogeny broth (LB) supplemented with chloramphenicol (25 mg ml^−1^) or kanamycin (25 mg ml^−1^), at 37°C with aeration. Prior to plant inoculations, *E. coli* Sakai was subcultured at 1:100 dilution into MOPS medium supplemented with amino acids and 0.2% glucose, termed rich‐defined RD‐MOPS glucose (Neidhardt *et al*., 1974) with antibiotics as required, and incubated with aeration for ~20 h at 18°C to pre‐adapt the bacteria to plant‐relevant growth temperatures. Cultures were diluted to OD_600_ of 0.02 (equivalent to 10^7^ CFU ml^−1^) in 0.5 × MS medium (Murashige and Skoog) without sucrose and diluted further as required. Long‐term stocks of bacteria were stored in 20% glycerol at −80°C. MacConkey agar was used for selection of *E. coli*, otherwise LB agar was used.

The plasmid‐borne GFP fluorescent reporter was generated previously with the *gyrA* promoter was fused to *gfp+*, termed p*gyrA‐gfp* (Holden *et al*., 2006). An equivalent red fluorescent reporter was generated by fusing the same promoter to a far‐red RFP, mKate (Shcherbo *et al*., 2007) derived from pNW725 (Marlow *et al*., 2014) and subcloned into pACYC184 using *Bam*HI and *Hind*III restriction enzyme sites. The *gyrA* promoter from *E. coli* was then cloned upstream of *mKate* using primers gyrA_H3 (5′‐CCCAAGCTTCAATATAGCCCAGACGCA) and gyrA‐BH1 (5′‐CGCGGATCCGCTATCCCTCTACTGTATCC) and termed p*gyrA‐rfp*. *E. coli* Sakai was transformed with the plasmids to produce two strains referred to as *E. coli* Sakai‐GFP and Sakai‐RFP. Stability of the GFP plasmid was tested by inoculating 3 × 2 μl drops of GFP‐Sakai diluted in 0.5 × MS to OD_600_ of 0.02 onto the abaxial epidermis of mOrg‐LTI‐benth or spinach leaves, seven replicated plants each, and maintained in the growth chamber for 12 days. The inoculated area was cut from the leaf with a cork borer, extracted in PBS following maceration using a pestle and mortar, the cells serially diluted 10‐fold in PBS and recovered on MacConkey agar plates either with or without chloramphenicol. A paired t‐test for means was used to compare the results ± antibiotic selection, at the 95% confidence level.

### Curli expression and production

The *csgBA* promoter from *E. coli* O157:H7 (Sakai) was cloned into the *Xba*I site in pKC026 using primers csgB.XbaI.F (5′‐CTCTAGATATTTACGTGGGTTTTAATACTTTGG) and csgB.XbaI.R (5′‐GGTCTAGAGTTGTCACCCTGGACCTGG), to generate a *csgBA‐gfp*+ transcriptional fusion plasmid, termed pAH004. *E. coli* Sakai transformed with pAH004 or pKC026 was incubated in LB at 37°C, 200 r.p.m. for ~18 h, and then subinoculated at 1:100 dilution into RD‐MOPS with glucose or glycerol (0.2%), or in MOPS supplemented with 40% (v/v) spinach leaf lysate and incubated statically at 18°C for 72 h. Triplicate samples of 200 μl and 1 ml were taken for fluorescence measurement in a black 96‐well microplate (GloMax‐Multi Detection System with blue optical kit, Promega), or for cell density measurement at OD_600_ respectively_._ Relative fluorescence units (RFU) were corrected for background fluorescence from the vector‐only control (pKC026) and plotted against the cell density measurements to obtain a nonlinear line of best fit, second‐order polynomial within 95% confidence intervals (prism5, graphpad). Elicitation of curli fibres was assessed on indicator plates, containing no‐salt lysogeny broth or M9 glucose medium supplemented with 40 μg ml^−1^ Congo red and 20 μg ml^−1^ Coomassie brilliant blue, solidified with 15 g L^−1^ agar, from three independent experimental replicates, with *Salmonella enterica* serovar Senftenberg (isolate 20070885) as a positive control. Colony colour was scored dark red (curli positive, e.g. for *S. enterica*), red, pink or white (negative control) as per Zhou *et al*. (2013).

### Plant material

*Nicotiana benthamiana* Domin was grown from seed stocks maintained at the James Hutton Institute. Seeds of spinach (*Spinacia oleracea* L. var. Amazon), lettuce (*Lactuca sativa* L. var. All Year Round, termed AYR) and tomato (*Solanum lycopersicum* L. var. Moneymaker) were obtained from Sutton Seeds, UK. Seeds were sown in commercial compost and maintained under glasshouse conditions with 16 h daylight, daytime temperature 26°C maximum, night‐time 22°C before transfer to an environmental cabinet with 16 h daylight, at a constant temperature of 18°C and 65–70% humidity prior to inoculation. Plants were used for experiments at around 4 weeks after sowing. Spinach leaf lysates were generated as described previously (Crozier *et al*., 2016), essentially by grinding fresh leaves from ~4 week old plants in liquid nitrogen, and clarifying by centrifugation and heat treatment at 50°C, prior to filtration. The lysates were used at 40% (v/v) final concentration.

mOrange‐LTI6b plasmids were generated by modification of the EGFP‐LTI6b marker of Kurup and coworkers (Kurup *et al*., 2005), were mobilized into *Agrobacterium* strain LBA4404 and used to transform *N. benthamiana* leaf segments as described by Horsch and associates (Horsch *et al*., 1985), with spectinomycin‐resistant plants (termed mOrg‐LTI‐benth) being regenerated and screened for expression by fluorescence microscopy. As the mOrange protein labels the plant cell plasma membrane, these plants were utilized to identify the position of intact plant cells in the absence of other staining methods.

### Measurement of internalization ability

*Escherichia coli* Sakai, grown as described above, was suspended in sterile distilled water at an OD_600_ of 0.02 (~10^7^ CFU ml^−1^) and then further diluted 1:100 000 to achieve ~10^2^ CFU ml^−1^, at a volume of 1 L per six plants. To inoculate leaves, the plants (still in their pots) were inverted and the aerial tissue submerged gently into the bacterial suspension for 30 s, and the plants placed upright and left for 1 h before sampling for the first time point. To inoculate the roots, the plant pots were submerged to ~1/3 height into the bacterial suspension, left for 1 h and removed. In both cases, sterile distilled water was used as a negative control. Samples were taken at time 0 (i.e. 1 hpi), day 5 and day 10 by aseptic removal of leaves or roots. The roots were washed in sterile distilled water to remove compost particles. The fresh weight of the samples was determined and they were stored briefly in 20 ml PBS prior to processing. To measure the total counts, five replicate samples were homogenized using a mortar and pestle in PBS, plated onto MacConkey agar with kanamycin in serial dilutions, incubated at 37°C and colonies enumerated the next day. To measure internalized counts, a second batch of five replicate samples was submerged in 20 ml 0.03% (w.v) calcium hypochlorite. The samples were incubated for 5 min at room temperature, with gentle shaking at 80 r.p.m. and then washed five times in PBS. The presence of any remaining surface‐associated *E. coli* Sakai was determined by placing each sample onto an agar dish containing MacConkey medium with kanamycin for ~10 s, termed ‘imprint plates’. The samples were then processed as for the total counts. Any samples with colonies on the imprint plates were discarded from subsequent analysis as incompletely surface‐sterilized, whereas those with no surface‐associated colonies were deemed to only contain internalized bacteria. Data analyses were conducted separately for the two inoculum levels (Genstat v16; VSNI Ltd., Hemel Hempstead, UK). As the data were highly skewed by the number of internalized samples where no bacteria were detected, two‐phase analyses were carried out. First, the data were rescored as presence/absence of bacteria and analysed using logistic regression. Then, the non‐responding samples were removed and unbalanced anova was used to test for differences between treatments in samples where bacteria were detected. Differences were considered significant at the 95% confidence level.

### Confocal microscopy

For imaging purposes, leaves were infiltrated with bacteria suspended in 0.5 × MS buffer at 10^7^ CFU ml^−1^ by pressure injection using a 1 ml syringe (without a needle) into the abaxial epidermis and the plants maintained in an environmental cabinet until observed. Leaf segments were infiltrated with sterile distilled water, to displace air from the apoplastic spaces between the spongy mesophyll cells, prior to mounting on microscope slides using double‐sided tape. The abaxial surface of the leaf was observed using a Nikon A1R confocal laser scanning microscope mounted on a NiE upright microscope fitted with an NIR Apo 40× 0.8W water dipping lens and GaAsP detectors. Images represent false‐coloured single sections, maximum intensity, 3D or orthogonal projections as indicated, produced using NIS‐elements AR software. GFP (green) and chlorophyll (blue) were excited at 488 nm with the emissions at 500–530 nm and 663–737 nm, respectively, and mOrange or mKate (RFP) were excited at 561 nm with emission at 570–620 nm (magenta). Where appropriate, leaves were infiltrated with an aqueous solution of calcofluor white M2R (Fluorescent Brightener 28; Sigma, St. Louis, Missouri, USA) at 0.1 mg ml^−1^ that was excited sequentially at 405 nm and the emission detected at 425–475 nm (cyan), and/or 5 mM Congo red (5 mM) imaged using the same settings as mOrange. DAPI (4′, 6‐diamidino‐2‐phenylindole), 1 μg ml^−1^, was imaged using the same settings as calcofluor. On occasion, laser light was directed through the tissue to the transmitted light detector to obtain a transmission image (grey). Isolated bacteria from an 18°C overnight culture were stained *in vitro* with dyes as above before being mounted under a coverslip on a cushion of 2% Oxoid No. 1 agar and observed using a CFI Plan Apochromat VC 60× WI lens.

### Determination of growth rates *in planta*

*Escherichia coli* Sakai were diluted to 10^3^ or 10^5^ CFU ml^−1^ in 0.5 × MS and 0.1 ml infiltrated into individual leaves, one per plant, by pressure injection as described above and the plants maintained in an environmental cabinet. Leaves were harvested at random from six plants at the designated time points, ground in 1 ml PBS and added to 5 ml buffered peptone water (BPW) with chloramphenicol. This was serially diluted, 10‐fold, to extinction with 3 × 1 ml replicates for each dilution. Visible growth was assessed following overnight incubation at 37°C and 10 μl spots were plated onto MacConkey agar with chloramphenicol and incubated overnight for confirmation of *E. coli* Sakai‐GFP. Colony growth was scored (+/−) and the MPN per ml of extract determined (Cochran, 1950), and this was multiplied by 6 to determine the number in the whole leaf. Using the six replicate measurements for *N. benthamiana* (four experiments), spinach and tomato (two experiments each), the estimates for most probable numbers of *E. coli* Sakai‐GFP bacteria, after log_10_ transformation, were analysed separately using a linear regression to estimate changes in population size during the first 20 days of the experiment (Genstat v16; VSNI Ltd.).

## Conflict of interest

None declared.

## Supporting information


**Fig. S1.** Staining of *Escherichia coli* O157:H7 Sakai bacteria *in vitro* with calcofluor white M2R, Congo red or DAPI.
