# Supplementary figures and images for: Differences in internalization and growth of Escherichia coli O157:H7 within the apoplast of edible plants, spinach and lettuce, compared with the model species Nicotiana benthamiana
Source: Microb Biotechnol. 2017 Feb 7;10(3):555–69. doi: 10.1111/1751-7915.12596 (PMC5404196; doi:10.1111/1751-7915.12596)

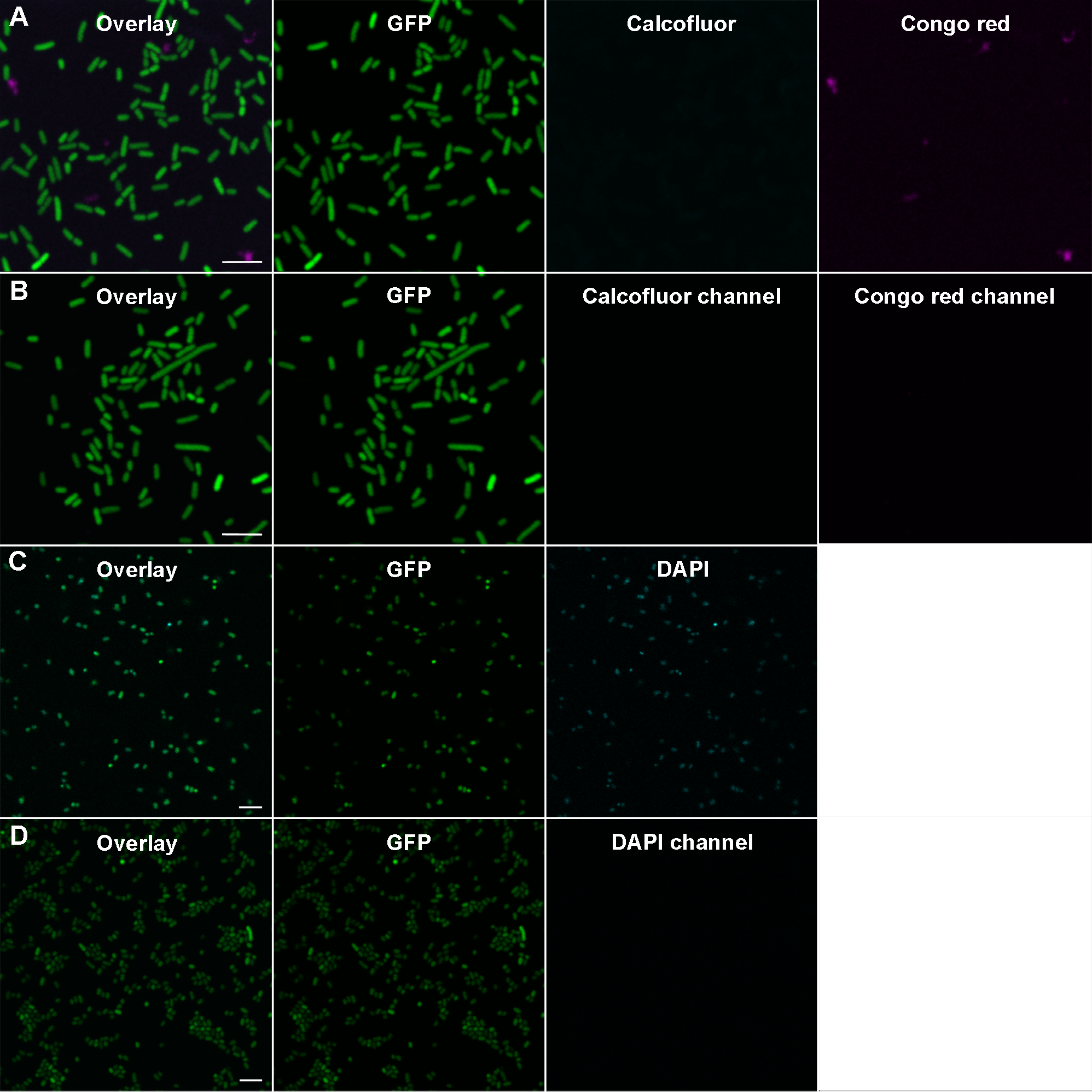

Supplement: Supplementary file 1 — Fig. S1. Staining of Escherichia coli O157:H7 Sakai bacteria in vitro with calcofluor white M2R, Congo red or DAPI. [file MBT2-10-555-s001.tif]
